# Treatment of cholangiocarcinoma by pGCsiRNA-vascular endothelial growth factor in vivo

**DOI:** 10.2478/abm-2024-0009

**Published:** 2024-04-30

**Authors:** Shenglin Lu, Jun Li

**Affiliations:** Jiangbei Branch of Zhongda Hospital of Southeast University, Nanjing, Jiangsu 210048, China

**Keywords:** cholangiocarcinoma, matrix metalloproteinase, mouse, treatment, vascular endothelial growth factor

## Abstract

**Background:**

The early diagnosis and treatment of cholangiocarcinoma may benefit from specific tumor markers to be used in clinical practice.

**Objectives:**

To investigate whether the pGCsiRNA-vascular endothelial growth factor (VEGF) can affect the onset and progression of cholangiocarcinoma and its possible mechanism using the targeted therapy of nude mouse model of cholangiocarcinoma with attenuated *Salmonella* carrying the plasmid pGCsiRNA-VEGF.

**Methods:**

The nude mouse model of cholangiocarcinoma was established by tail vein injection of QBC939 cells and given attenuated *Salmonella* carrying the plasmid pGCsiRNA-VEGF. One month later, the tumor volume of nude mice was observed, and the tumor growth curve was plotted. The harvested tumors were weighed and detected for tissue structural changes and cell death status by hematoxylin–eosin staining. The protein and mRNA expressions of VEGF, matrix metalloproteinase 2 (MMP2), and MMP9 were detected by Western blotting and PCR, respectively.

**Results:**

The tumor volume and weight of the pGCsiRNA-VEGF group were significantly smaller than those of the mock and the si-scramble groups (*P* < 0.05). The expressions of VEGF, MMP2, and MMP9 at the transcriptional and translational levels were inhibited by pGCsiRNA-VEGF. PGCsiRNA-VEGF promoted tissue apoptosis and destroyed the tissue structure.

**Conclusions:**

In vivo silencing of VEGF can affect cell survival and inhibit cell migration, invasion, and development, probably by enhancing apoptosis and inhibiting the expressions of MMP2 and MMP9.

The early diagnosis and treatment of cholangiocarcinoma, a common biliary tract malignancy characterized by a high degree of malignancy, insidious onset, and having no specific tumor markers in clinical practice, is highly difficult [1]. Meanwhile, no effective chemotherapeutic drug has been used, and cholangiocarcinoma has neural invasion and skip metastasis, with a high recurrence rate after treatment [2]. As a result, the 3-year survival rate of patients with cholangiocarcinoma is lower than 11% [3]. As demonstrated by a large number of experiments, the vascular endothelial growth factor (VEGF) is highly expressed in cholangiocarcinoma, and its expression is strongly positive in 41.7% of the patients and weakly positive in 27.8% of the patients [4]. Previously, the expressions of VEGF and VEGF receptor 2, i.e., kinase domain-containing receptor (KDR), were analyzed using the immunohistochemical assay in 60 cases of cholangiocarcinoma tissues and 40 cases of normal bile duct epithelial tissues. The positive rates of VEGF and KDR were 88% (53/60) and 85% (51/60), respectively, in 60 cases of cholangiocarcinoma tissues, and their expression levels were up-regulated with the increase in the tumor pathological stage and cellular grade. In contrast, no expression of VEGF and KDR was found in 40 cases of the normal bile duct epithelial tissues [5]. It can be inferred that VEGF may play a role in the neovascularization of cholangiocarcinoma through binding to KDR in bile duct epithelial cells, thus facilitating the growth and metastasis of cholangiocarcinoma [6]. Therefore, it is of importance to further study the role of VEGF in the development mechanism of cholangiocarcinoma [7, 8].

In this study, the expression of VEGF was inhibited by the RNA interference (RNAi) technique, and the plasmid pGC-siRNA-VEGF expressing siRNA-VEGF was constructed. Then, the effect of attenuated *Salmonella* carrying the plasmid pGCsiRNA-VEGF on nude mice with cholangiocarcinoma was explored. The findings provide a reliable theoretical basis for the gene therapy of cholangiocarcinoma.

## Methods

This project was approved by Jiangbei Branch of Zhongda Hospital of Southeast University (certificate of approval No. JBZHSU-Animal-202103004).

### Establishment of nude mouse xenograft models of cholangiocarcinoma

Cholangiocarcinoma QBC939 cells were injected subcutaneously into the back of nude mice (1 × 10^7^ cells/mouse). When the tumor grew to about 5 mm in diameter, it was freshly harvested and cut into pieces (1 mm × 1 mm × 1 mm). The nude mice were anesthetized with 2% pentobarbital, and the abdomen was disinfected and cut open along the linea alba. Then the tumor tissue was inoculated closely near the bile duct, and finally the abdomen was closed. After surgery, the mice were fed for 4–6 weeks.

### Construction of plasmid pGCsiRNA-VEGF

**Figure 1.**
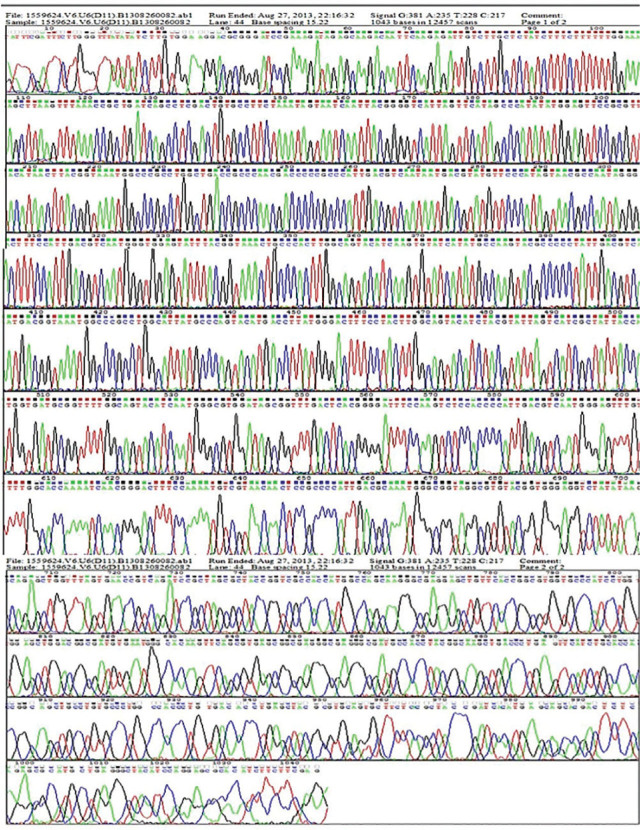
Sequencing results of pGCsiRNA-VEGF containing U6 promoter. The results showed that 6.3 kb linearized vectors and 63 bp oligonucleotide fragments were obtained. pGCsiRNA, VEGF, vascular endothelial growth factor.

VEGF oligonucleotides synthesized by Sangon Biotech Co., Ltd. (Shanghai, China) were annealed and ligated into the pGCSilencerTMneo2.0-U6/GFP-siRNA expression vector, and positive clones were screened out after transformation (**Figure 1**). The plasmids were extracted in accordance with the instructions of the kit (Thermo Fisher Scientific, USA) and identified by digestion with *Hin*dIII and *Bam*HI. Then the digested products were identified by 2% agarose electrophoresis, and the results showed that 6.3 kb linearized vectors and 63 bp oligonucleotide fragments were obtained. The synthesized plasmids were sent to Shanghai Invitrogen Biotechnology Co., Ltd. (China) for sequencing.

### Infection of cholangiocarcinoma nude mice with attenuated *Salmonella* carrying the plasmid pGCsiRNA-VEGF

Twenty-one nude mice with cholangiocarcinoma were randomly divided into 3 groups (mock group, pGCsiRNA-VEGF group, and si-scramble group), with 7 mice in each group. The mice in the mock group were perfused with PBS, those in the pGCsiRNA-VEGF group were infected by an oral perfusion with attenuated *Salmonella* carrying the plasmid pGCsiRNA-VEGF at 1 × 10^4^ CFU/mL, and those in the si-scramble group were infected by an oral perfusion with attenuated *Salmonella* carrying plasmid pGCsiRNA. The eating, drinking, and mental status of mice was observed in each group, and the tumor growth status was observed and recorded every other day after treatment.

### Histopathological examination

On Day 28 of treatment, the nude mice were sacrificed in each group. The tumors were harvested, weighed, and photographed. Then the tumor tissues were fixed in 10% formalin solution, embedded in paraffin, and sectioned. After deparaffinization and hematoxylin–eosin (HE) staining, microscopy was performed to observe the effects of different treatments on tissues of mice.

### Detection of protein expressions of VEGF, matrix metalloproteinase 2 (MMP2), and MMP9 by Western blotting

About 1–3 pieces of tumor tissue (about 0.5 g) were harvested from each mouse, washed with normal saline 2–3 times, and prepared into homogenates. The protein was extracted with RIPA lysis buffer, subjected to electrophoresis (30 μg/well), and transferred onto PVDF membranes. Then the membrane was sealed with 5% skim milk at room temperature for 1 h and incubated with anti-VEGF, MMP2, and MMP9 antibodies (1:1,000) at 4°C overnight. After washing, the membrane was incubated again with horseradish peroxidase-labeled goat anti-rabbit IgG secondary antibody (1:10,000) at room temperature for 30 min. Finally, color was developed by ECL, images were acquired, and the intensity of protein bands was analyzed with Image Lab software (Bio-Rad, USA).

### qRT-PCR

One piece of tumor tissue (about 1 g) was harvested from each mouse, washed with normal saline 2–3 times, and prepared into homogenates. Total RNA was extracted with 1 mL of TRIzol (Invitrogen, USA) and its concentration was measured. Then, 500 ng of total RNA was reversely transcribed into cDNA according to the instructions of the PrimeScript RT kit (Takara, Japan), and PCR was performed on a fluorescence quantitative PCR instrument (Bio-Rad, USA) using the qRT-PCR kit (Vazyme, Nanjing, China).

### Statistical analysis

Prism software (GraphPad, USA) was used for statistical analysis, and the results were described as mean ± standard deviation. Comparison was conducted between the 2 groups by the Student’s *t* test, and *P* < 0.05 was considered statistically significant. All tests were 2-tailed, and all test results had 3 biological replicates.

## Results

### Inhibitory effect of plasmid pGCsiRNA-VEGF on cholangiocarcinoma xenografts

**Figure 2.**
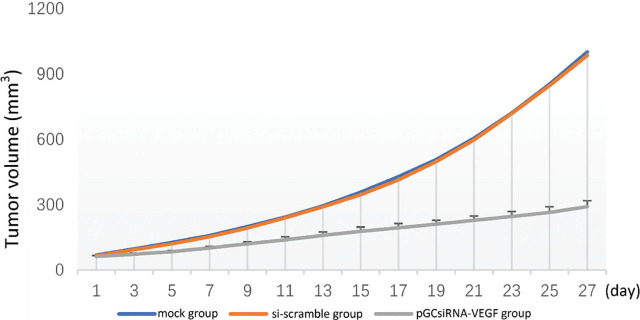
Tumor growth curves. The tumor volume was smaller in the pGCsiRNA-VEGF group than that in the mock group and the si-scramble group on Day 27 of treatment. VEGF, vascular endothelial growth factor.

**Figure 3.**
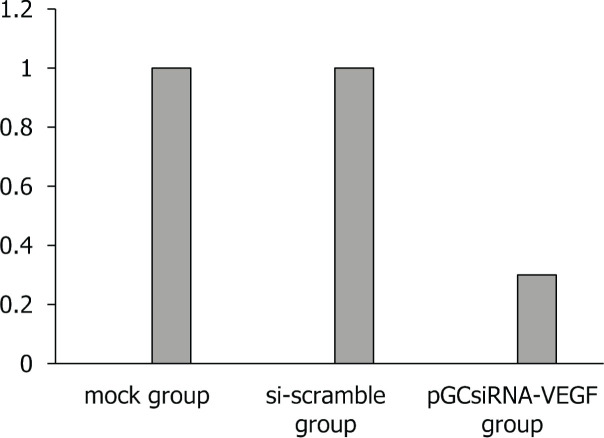
Tumor weights. On Day 28 of treatment, the tumor weight was smaller in the pGCsiRNA-VEGF group than that in the mock group and the si-scramble group. VEGF, vascular endothelial growth factor.

**Figure 4.**
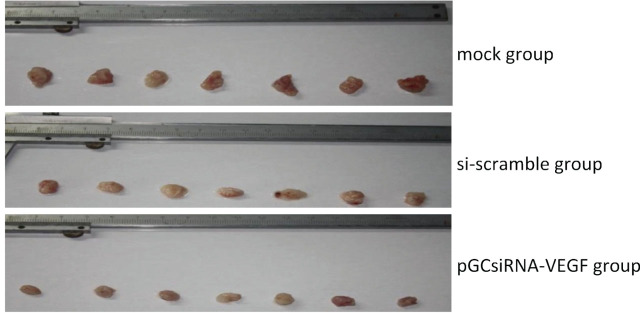
Tumor volumes. Tumor growth was inhibited in nude mice. VEGF, vascular endothelial growth factor.

Tumors, with a diameter of about 5 mm, formed on the right side of nude mice about 2 weeks after inoculation with QBC939 cells, suggesting the successful establishment of the mouse xenograft model of cholangiocarcinoma. After grouping, the treatment was initiated (Day 1), the tumor growth status was observed every other day, and the tumor growth curve of each group was plotted (**Figure 2**). The tumor volume was measured every other day after treatment. The tumor volume was smaller in the pGCsiRNA-VEGF group than that in the mock group and the si-scramble group on Day 27 of treatment (*P* < 0.05). On Day 28 of treatment, the nude mice were sacrificed. The tumor weight was smaller in the pGCsiRNA-VEGF group than that in the mock group and the si-scramble group (*P* < 0.01). Collectively, the tumor growth in nude mice was inhibited (**Figures 3 and 4**).

### Changes in protein expressions of VEGF, MMP2 and MMP9 in tumor tissues

**Figure 5.**
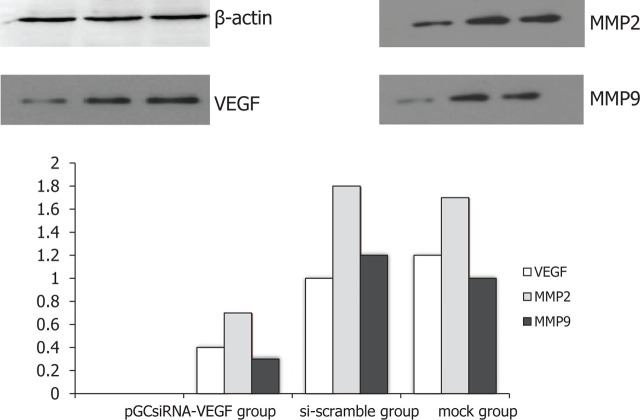
Protein expressions of VEGF, MMP2 and MMP9. The protein expressions of VEGF, MMP2, and MMP9 declined in pGCsiRNA-VEGF group compared with those in the mock and si-scramble groups. MMP, matrix metalloproteinase; VEGF, vascular endothelial growth factor.

The protein expressions of VEGF, MMP2 and MMP9 significantly decreased in pGCsiRNA-VEGF group compared with those in the mock and si-scramble groups (*P* < 0.01) (**Figure 5**).

### Changes in mRNA expressions of VEGF, MMP2, and MMP9 in tumor tissues

**Figure 6.**
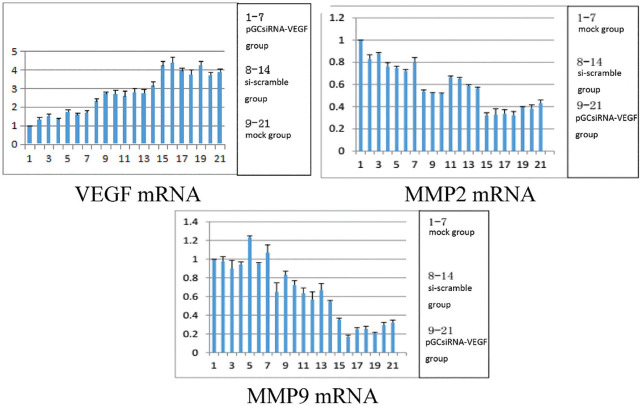
Changes in mRNA expressions of VEGF, MMP2, and MMP9 in tumor tissues. The mRNA expressions of VEGF, MMP2, and MMP9 were significantly lower in the pGCsiRNA-VEGF group than those in the mock group and the si-scramble group. MMP, matrix metalloproteinase; VEGF, vascular endothelial growth factor.

The mRNA expressions of VEGF, MMP2, and MMP9 were significantly lower in the pGCsiRNA-VEGF group than those in the mock and si-scramble groups (*P* < 0.05) (**Figure 6**).

### Histopathological examination results

**Figure 7.**
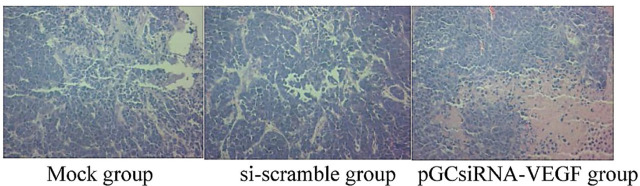
Morphological changes of tumor tissues. There was obvious apoptosis of tumor tissues, a large number of cells died, the tissue structures mostly disappeared, karyopyknosis was found, a large number of apoptotic cells were dispersed around the tissues, and the cells lost their normal morphology in pGCsiRNA-VEGF group compared to those in the mock and si-scramble groups. VEGF, vascular endothelial growth factor.

It was observed by HE staining that in the pGCsiRNA-VEGF group, there was obvious apoptosis of tumor tissues, a large number of cells died, the tissue structures mostly disappeared, karyopyknosis occurred, many apoptotic cells were dispersed around the tissues, and the cells lost their normal morphology (**Figure 7**).

**Figure 8.**
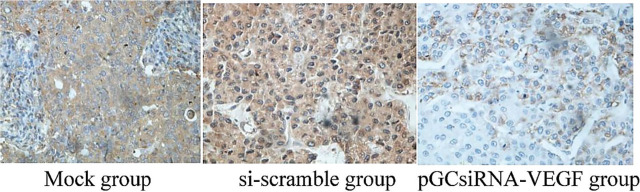
VEGF expressions of the different groups. The protein expression of VEGF was significantly suppressed in the pGCsiRNA-VEGF group compared with that in the mock group and the si-scramble group. VEGF, vascular endothelial growth factor.

The protein expression of VEGF was significantly suppressed in the pGCsiRNA-VEGF group compared with that in the mock and si-scramble groups (**Figure 8**). Taken together, the expressions of VEGF, MMP2, and MMP9 at the transcriptional and translational levels were inhibited by pGCsiRNA-VEGF. PGCsiRNA-VEGF promoted tissue apoptosis and destroyed the tissue structure.

## Discussion

The early diagnosis and treatment of cholangiocarcinoma is highly difficult [9, 10]. This cancer has a high recurrence rate after treatment, and the 3-year survival rate of patients is lower than 11% [11]. Previously, cholangiocarcinoma was mainly treated with surgery and liver transplantation [12]. However, patients with cholangiocarcinoma have been generally in the late stage when clinically diagnosed, so the prognosis remains poor despite effective lesion clearance, and the condition of disease cannot be effectively relieved by postoperative radiotherapy and chemotherapy [13]. After transfection, oncogenes significantly increase the activity of angiogenesis by raising the VEGF expression, while neovascularization plays an important role in tumor growth, invasion, and metastasis [14]. Then VEGF binds VEGFR on vascular endothelial cells and tumor cell membranes to induce vascular endothelial cell proliferation and stimulate increased vascular permeability, thereby resulting in tumor growth and metastasis [15, 16].

With high tumor-targeting ability and low pathogenicity, attenuated *Salmonella* has been used as the plasmid vector by many researchers [17]. In particular, *Salmonella* is injected via the tail vein in nude mouse experiments, achieving a good therapeutic effect [18]. In this study, the results of in vivo studies showed that attenuated *Salmonella* carrying plasmid pGCsilencer-U6/Neo/GFP had a definite effect in the treatment of cholangiocarcinoma xenografts in nude mice. On Days 7–9 of treatment, the tumor volume shrank in the pGCsiRNA-VEGF group compared with that in the mock group and the si-scramble group (*P* < 0.05). On Day 27 of treatment, the tumor volume in the pGCsiRNA-VEGF group was significantly smaller than that in the mock group and the si-scramble group (*P* < 0.01). It was observed by HE staining that there was obvious apoptosis of tumor tissues, a large number of cells died, the tissue structures mostly disappeared, karyopyknosis was found, a large number of apoptotic cells were dispersed around the tissues, and the cells lost their normal morphology in the pGCsiRNA-VEGF group as compared to the mock group and the si-scramble group, suggesting significant efficacy in the pGCsiRNA-VEGF group. Moreover, the results of the immunohistochemical assay revealed that the protein expression of VEGF was significantly lower in the pGCsiRNA-VEGF group than that in the mock group and the si-scramble group, consistent with the results of PCR and Western blotting. The above findings demonstrated that the plasmid pGCsilencer-U6/Neo/GFP displays a good therapeutic effect. The detection results of apoptosis- and migration-related genes were consistent with those of in vitro experiments, verifying that both apoptosis and migration of tumor cells were weakened [19, 20].

In this study, the plasmid pGCsilencer-U6/Neo/GFP was successfully constructed for the first time. It was proved by in vivo experiments that the pGCsilencer-U6/Neo/GFP could restrain the proliferation, migration, and invasion of cholangiocarcinoma while promoting the apoptosis of tumor cells. By targeted therapy with pGCsilencer-U6/Neo/GFP, VEGF was silenced, thus reducing the expressions of MMP2 and MMP9. It can be inferred that VEGF negatively regulates MMP2 and MMP9.

In conclusion, it was verified by in vivo experiments that pGCsilencer-U6/Neo/GFP could inhibit the proliferation, migration, and invasion of cholangiocarcinoma, while promoting the apoptosis of tumor cells, whose mechanism may be related to the ability of VEGF silencing to enhance apoptosis and inhibit the expressions of MMP2 and MMP9. Attenuated *Salmonella* can serve as a vector for gene therapy to exert a significant therapeutic effect on deep tumors.
